# Characterization of the complete mitochondrial genome of cape elephant shrew, *Elephantulus edwardii*

**DOI:** 10.1080/23802359.2018.1483759

**Published:** 2018-07-03

**Authors:** Chang-Zhong Liu, Lei Wang, Xiao-Jing Xia, Jin-Qing Jiang

**Affiliations:** College of Animal Science and Veterinary Medicine, Henan Institute of Science and Technology, Xinxiang, Henan Province, China

**Keywords:** Cape elephant shrew, Elephantulus edwardii, mitochondrial genome, phylogenetic analysis

## Abstract

In this study, the complete mitochondrial genome of cape elephant shrew, *Elephantulus edwardii,* was determined through sequencing of PCR fragments. The complete mitochondrial genome of *E. edwardii* was 16,552 bp in length and and encoded 13 protein-coding genes, 22 transfer RNA (tRNA) genes, and two ribosomal RNA genes. The overall nucleotide composition is: 32.7% A, 29.5% T, 25.0% C, and 12.9% G, with a total G+C content of 37.9%. By phylogenetic analysis using Bayesian method, *E. edwardii* showed the closest relationship with another *Elephantulu*s speceis (Genbank accession: AB096867.1).

The cape elephant shrew, *Elephantulus edwardii*, is neither closely related to shrews nor to rodents (mice), but belong to the order Macroscelidea (family Macroscelididae) with currently 17 species in four genera distributed in Africa and is fundamental to evaluating its recognition as vulnerable in the IUCN Red List.

In this study, the specimen was collected from the Matsikamma mountain (18°49.518′ E, 31°44.203′S), Africa. The total DNA was extracted from muscular tissue, using the commercial Animal Tissues Genomic DNA Extraction Kit (Solarbio, BJ, CN) following the manufacturer’s instructions, and then used as the template for polymerase chain reaction (PCR) amplifications. Fifteen pairs of primer were designed based on mitogenome of *Elephantulus sp.* VB001 (Genbank accession: AB096867.1). The muscular tissue was stored at −80 °C and extracted genomic DNA was sotred at −20 °C in the Zoology comprehensive laboratory from College of Animal Science and Veterinary Medicine. The isolated DNA was sent to sequencing company (BGI Tech, Shenzhen, China). The amplified products were sequenced using the amplification primers. All sequencing was done by a commercial sequencing service (BGI Tech, Shenzhen, China). Some small number of PCR products that could process complex secondary structures or high A+T content, were cloned into the PMD-19T vector (TaKaRa, Dalian), then transformed to JM109 competent cell (TaKaRa, Dalian) for sequencing. All sequencing sequences were assembled with the program Seqman in the DNASTAR package (Burland 1999).

Protein coding genes were predicted using ORF Marker tool in UGENE (Okonechnikov et al. 2012) under the vertebrate mitochondrial genetic code. Protein-coding regions and ribosomal RNA genes were also annotated manually and confirmed by comparison to the mitogenome of *Elephantulus sp.* (Genbank accession: AB096867.1) that available in Genbank. The transfer RNA (tRNA) genes were identified and assigned putative secondary structures using the program tRNAscan-SE (Lowe et al. 1997) or by manually identifying potential secondary structures and anticodon sequences through visual inspection. The graphical map of the complete mitochondrial genome was drawn using the online sofware OrganellarGenomeDRAW (Lohse et al. 2007).

The complete mitogenome of *Elephantulus edwardii* (Genbank accession: MH252335) is a closed-circular molecule of 16,522 bp in length, which is a little shoter than mitogenome of *Elephantulus sp.* VB001. It presents the typical set of 37 genes observed in metazoan mitogenomes, including 13 PCGs (*cox*1-3, *co*b, nad1-6, *nad*4L, *atp*6, and *atp*8), 22 tRNA genes (one for each amino acid, two each for Leucine and Serine), two genes for ribosomal RNA subunits (*rrn*S and *rrn*L).

For phylogenetic analysis assessing the relationship of this mitogenome, we selected other 33 Eutheria mitogenomes downloaded from GenBank. The genome-wide alignment of all mt genomes was done by HomBlocks (Bi et al. 2018), resulting in 20,840 characters in total, including almost all whole or partial PCGs and rRNA genes. The whole genome alignment was analyzed by PhyloBayes ver. 3.3 (Lartillot et al. 2009) under the GTR+G model. Four independent MCMC analyses were run for 10,000 cycles in PhyloBayes. Convergence was checked based on time-series plots of the likelihood scores using Tracer (http://tree.bio.ed.ac.uk/software/tracer/). The first 25% cycles were discarded as burn-in, and the remaining trees were summarized to obtain Bayesian posterior probabilities (BPPs). The resulting tree was represented and edited using FigTree v1.4.1 (http://www.umiacs.umd.edu/∼morariu/figtree/). As shown in Figure. 1, the phylogenetic positions of these 34 mt genomes were successfully resolved with full BPPs supports across almost all nodes. As expected, *Elephantulus edwardii* showed the closest relationship with *Elephantulus sp.* VB001. And they clustered as Macroscelididae clade with *Macroscelides proboscideus*.

**Figure 1.**
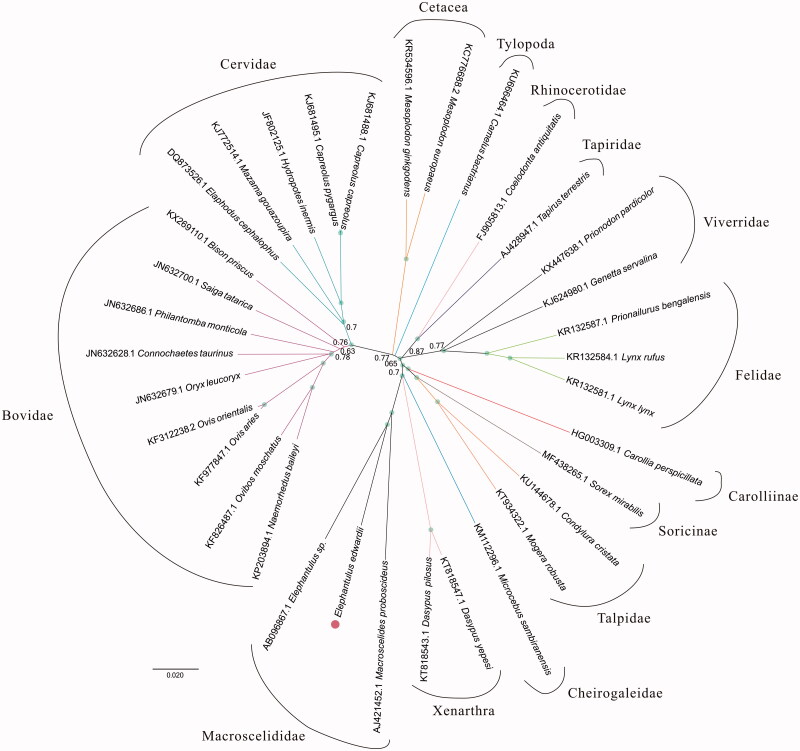
Phylogenetic relationships among 34 Eutheria mt genomes. This tree was drawn without setting of an outgroup. Nodes exhibit 100% posterior probability (PP) were labelled by green points.
